# The Influence of Obesity on Pain and Function in Knee Osteoarthritis: Comparison of Body Mass Index With Seven Knee Function Scales and Two Pain Scales

**DOI:** 10.7759/cureus.24304

**Published:** 2022-04-20

**Authors:** Deniz Gurler

**Affiliations:** 1 Orthopedics and Traumatology, Samsun Education and Research Hospital, Samsun, TUR

**Keywords:** knee osteoarthritis (koa), obesity-related illnesses, body mass index, pain scales, knee scales

## Abstract

Instruction: Obesity is a health problem that is rapidly increasing both in local societies and internationally. It is well known that obesity has a risk relationship with many different diseases. The scale for obesity is Body Mass Index (BMI), which has been widely accepted worldwide for many years. The relationship between BMI and disease is a frequently studied topic. This study aimed to evaluate and measure knee function and pain in patients with knee osteoarthritis.

Materials and Methods: A total of 100 patients in radiologically advanced stage (Kellgren/Lawrence grade 3-4) who were scheduled for knee arthroplasty were administered seven knee osteoarthritis scales (Timed up and Go (TUG), American Knee Society Score (AKSS), the Lequesne Knee Index, Knee injury and Outcome Subtotal Pain Score (KOOS-PS), Western Ontario and McMaster Universities Index (WOMAC), Oxford Knee Score, and International Knee Documentation Committee (IKDC)), and two pain scales, the McGill Pain Questionnaire and a visual analog pain scale (VAS), which were completed simultaneously on the same form. Data that did not show a normal distribution were analyzed with Spearman and Kendall correlation tests.

Results: The mean age of the 100 consecutive patients, 92% of whom were female, was 65.2 years (48-81 years). There was a strong correlation between BMI and all functional knee scales, but no significant association was found between pain scales and BMI.

Conclusion: In our hypothesis, we expected that all functional and pain scales would moderately or strongly correlate with BMI. However, while a strong correlation with the functional pain scales is an expected result, the expected strong positive correlation between pain scales and BMI was not found in the study.

## Introduction

Obesity is a severe health problem in terms of mortality and morbidity. Its importance is increasing day by day worldwide. Particularly in industrialized countries, solutions are sought for economic reasons since it represents a significant item of expenditure in health care.

In the early years of the Industrial Revolution, only the wealthy elite had easy access to food. However, this opportunity spread to the middle-income classes after World War II, especially in developed Western societies. As technological developments increased the efficiency of food production, food became easily accessible for the lower economic classes as well. These improvements in the processing of food and easy availability also paved the way for rapid weight gain in populations and, consequently, gave rise to an obesity epidemic. The role of fast food in the spread of obesity is undeniable. Regardless of economic or social class, obesity is now considered a health problem in developed or developing countries rather than a problem of dietary habits. It is thus ironic that, according to the United Nations, 811 million people worldwide will suffer from hunger in 2020 [1].

The link between obesity and other health issues has been studied for many years. Obesity is a significant risk factor for increased morbidity and mortality, especially cardiovascular disease, diabetes, chronic diseases such as liver and kidney disease, sleep apnea, and depression [2]. Obesity has also been described as a risk factor for knee osteoarthritis [3].

Body mass index (BMI) is the most popular weight index to assess obesity today. BMI is a scale calculated based on height and weight written and analyzed by Ancel Keys in 1972 [4,5]. Although there are problems with measurement and data collection [6], BMI remains the most common scale for obesity assessment and treatment planning.

## Materials and methods

The study was conducted prospectively between January and October, 2015, at the Health Sciences University, Samsun Training And Research Hospital, Samsun, Turkey. The study was approved by the Republic of Turkey Ministry of Health Samsun Education and Research Hospital Scientific Research Evaluation Committee (approval number 33646832/900, dated December 19, 2014). Patients scheduled for knee arthroplasty were evaluated at the last preoperative visit according to the terms of the study. One hundred sixty-four patients scheduled for surgery were enrolled in the study. Patients with cerebrovascular disease and congenital or acquired advanced lower extremity deformities were excluded from the study. Of the 164 patients, 124 met the requirements for the study. One hundred and eight patients agreed to participate in the study and of these, 100 patients completed the form in full. Participants read and signed the informed consent form before the examination. Patient information was verified from the hospital computer registry.

This study aimed to evaluate knee osteoarthritis with different scales and the relationship with BMI. We applied seven scales and scores for knee osteoarthritis and two pain scales (combined into a single form) to 100 patients with radiologically advanced stage knee osteoarthritis (Kellgren/Lawrence grade 3-4). These seven scales and scores are Timed Up and Go (TUG), American Knee Society Score (KSS), LeQuesne Knee Index, Knee Injury and Osteoarthritis Outcome-Pain Subtotal Score (KOOS-PS), Western Ontario and McMaster Universities Osteoarthritis Index (WOMAC), Oxford Knee Score (OKS), and International Knee Documentation Committee (IKDC). The McGill Pain Questionnaire and Visual Analog Pain Scale (VAS) were also queried on this form. The patients' functional and pain abilities were recorded. All seven scales and two pain scales in a single form were administered to patients in the preoperative preparation phase. The patients' physical abilities were recorded with knee scales, and the correlation of these data with BMI data was studied. BMI was calculated from the recorded height and weight in the questionnaire using Microsoft Excel (Microsoft Corporation, Redmond, Washington, United States). The variables obtained from these data tables (values of all scales and BMI) were transferred to the statistical program.

The data were analyzed using the Jamovi 2.2.2 statistical analysis program (Released October 2021). The study data were analyzed and found to be non-normally distributed (determined with the Shapiro-Wilk test). Since the scale values did not show a normal distribution, we used Spearman's rank coefficient of correlation and Kendall's τ coefficient, which are nonparametric correlation tests, to correlate the BMI and the scales (p <.001).

## Results

The mean age was 65.2 years (48-81 years), and the sex distribution was 92 female and eight male patients. Radiographs of all patients showed Kellgren/Lawrence grade 3-4, and all patients were selected among those scheduled for knee arthroplasty. The descriptive statistical results of the data used in the study are shown in Table 1.

**Table 1:** Descriptive statistical results of the data used in the study. BMI: Body Mass Index; TUG: Timed Up and Go; LeQuesne: LeQuesne Knee Index; KOOS-PS: Knee Injury and Osteoarthritis Outcome-Pain Subtotal Score; WOMAC: Western Ontario and McMaster Universities Osteoarthritis Index; OKS: Oxford Knee Score; KSS: American Knee Society; IKDC: International Knee Documentation Committee; VAS: Visual Analog Pain Scale; McGill: McGill Pain Questionnaire,

	Age	BMI	TUG	LeQuesne	KOOS-PS	WOMAC	OKS	KSS	IKDC	VAS	McGill
N	100	100	100	100	100	100	100	100	100	100	100
Mean	65.2	32.0	10.2	12.7	15.0	33.7	30.6	64.3	52.4	7.37	38.0
Median	64.5	32.5	9.90	13.0	15.0	31.8	30.5	65.0	52.0	7.65	35.0
Standard deviation	8.01	4.53	1.86	2.37	3.80	9.29	5.98	6.04	10.2	1.47	9.59
Minimum	48	22.6	7.30	9	8	20.8	20	48	35.5	4.30	23
Maximum	81	41.6	14.0	18	21	55.2	42	75	70.0	9.70	61
Shapiro-Wilk W	0.974	0.983	0.948	0.946	0.948	0.936	0.963	0.964	0.950	0.951	0.935
Shapiro-Wilk p	0.048	0.208	< .001	< .001	< .001	< .001	0.007	0.007	< .001	< .001	< .001

After statistical analysis, a strong correlation was found between functional knee scales and BMI for all parameters. Although the OKS had the lowest scores, the correlation was significant (τ=0.530, p=0.001). The strong positive correlations exhibited by these scales are also statistically supported. This result is consistent with our hypothesis that the functional knee scales of patients with knee osteoarthritis are related to obesity. However, we found no significant correlation between the pain scales and obesity. Correlation between BMI and pain scales Spearman and Kendall analysis results: McGill Pain Index ρ=0.087 (p=0.388), τ=0.065 (p=0.349), VAS ρ=0.007 (p=0.942) τ=0.008 (p =0.912). The correlation between the functional knee scales and the pain scales with BMI is shown in Table 2.

**Table 2:** Correlation of functional scales and pain scales with BMI. Note: Significant correlation between BMI and Function Scales (* p < .05, ** p < .01, *** p < .001) BMI: Body Mass Index; TUG: Timed Up and Go; LeQuesne : LeQuesne Knee Index; KOOS-PS: Knee Injury and Osteoarthritis Outcome-Pain Subtotal Score; WOMAC: Western Ontario and McMaster Universities Osteoarthritis Index; OKS: Oxford Knee Score; KSS: American Knee Society; IKDC: International Knee Documentation Committee; VAS: Visual Analog Pain Scale; McGill: McGill Pain Questionnaire,

		BMI	TUG	LeQuesne	KOOS-PS	WOMAC	OKS	KSS	IKDC	VAS	McGill
BMI	Spearman's rho	—									
	p-value	—									
	Kendall's Tau B	—									
	p-value	—									
TUG	Spearman's rho	0.857***	—								
	p-value		—								
	Kendall's Tau B	0.659***	—								
	p-value		—								
LeQuesne	Spearman's rho	-0.658***	-0.566***	—							
	p-value			—							
	Kendall's Tau B	-0.484***	-0.415***	—							
	p-value			—							
KOOS-PS	Spearman's rho	-0.685***	-0.666***	0.499***	—						
	p-value				—						
	Kendall's Tau B	-0.464***	-0.485***	0.361***	—						
	p-value				—						
WOMAC	Spearman's rho	0.998***	0.858***	-0.659***	-0.686***	—					
	p-value					—					
	Kendall's Tau B	0.980***	0.672***	-0.493***	-0.476***	—					
	p-value					—					
OKS	Spearman's rho	-0.723***	-0.599***	0.531***	0.470***	-0.718***	—				
	p-value						—				
	Kendall's Tau B	-0.530***	-0.411***	0.401***	0.325***	-0.535***	—				
	p-value						—				
KSS	Spearman's rho	-0.812***	-0.722***	0.529***	0.535***	-0.807***	0.539***	—			
	p-value							—			
	Kendall's Tau B	-0.623***	-0.542***	0.386***	0.381***	-0.629***	0.386***	—			
	p-value							—			
IKDC	Spearman's rho	-0.913***	-0.853***	0.651***	0.650***	-0.914***	0.673***	0.790***	—		
	p-value								—		
	Kendall's Tau B	-0.711***	-0.657***	0.473***	0.448***	-0.729***	0.485***	0.591***	—		
	p-value								—		
VAS	Spearman's rho	0.007	0.051	-0.100	0.033	0.015	0.086	0.040	-0.041	—	
	p-value	0.942	0.613	0.324	0.747	0.884	0.393	0.694	0.685	—	
	Kendall's Tau B	0.008	0.038	-0.066	0.022	0.013	0.054	0.025	-0.032	—	
	p-value	0.912	0.579	0.361	0.753	0.853	0.445	0.727	0.644	—	
McGill	Spearman's rho	0.087	0.186	-0.159	-0.001	0.094	-0.078	-0.069	-0.101	0.319**	—
	p-value	0.388	0.063	0.114	0.994	0.352	0.438	0.492	0.320	0.001	—
	Kendall's Tau B	0.065	0.127	-0.117	0.002	0.072	-0.059	-0.049	-0.069	0.218**	—
	p-value	0.349	0.068	0.110	0.974	0.307	0.404	0.490	0.316	0.002	—

## Discussion

Obesity is a health problem that affects the entire world, with mortality and morbidity rates increasing daily. More than one billion people worldwide are obese (BMI ≥ 30.0) - 650 million adults, 340 million adolescents, and 39 million children as of March 2022 [7]. The findings suggest that obesity will not disappear in the foreseeable future and that there will be significant problems related to obesity [8]. Although the social and economic backgrounds of obesity are not the main topic of our study, the evolutionary and sociological causes of obesity deserve to be examined. BMI has been used to assess obesity for many years. Therefore, we used BMI to determine the presence and severity of obesity. There is a significant risk of osteoarthritis in patients with a BMI≥ 25 over 40 years. An increase in BMI of five units results in a 39% increase in the risk of osteoarthritis [9]. However, a significant problem with BMI is the calculation of BMI based on self-reports in some records. Self-reported BMI data are lower than actual measurements [6]. Therefore, we measured and recorded the data instead of using self-reported patients' weight and height values.

Obesity increases the risk of knee osteoarthritis and knee arthroplasty [10]. Many studies show a strong association between BMI and osteoarthritis. While obesity has not been shown to affect the progression of osteoarthritis [11], some publications report otherwise; therefore, this issue is still open for new research. Obesity is also known to increase the risk of other systemic diseases. Although a significant association with osteoarthritis has been found in postmenopausal obese women, this association has only been observed in knee osteoarthritis. Obesity is seen to affect blood lead levels in postmenopausal women, which affects knee osteoarthritis [12]. In osteoarthritis, insulin-like growth factor-1 (IGF-1) is significantly increased. This level is known to be related to obesity. Radiographic findings in patients with osteoarthritis may not be related to pain and functional limitations. Although 37% of those over 60 years of age in the United States had radiographic evidence of knee osteoarthritis, only 12% had symptoms [8]. Osteoarthritis of the knee is 4.59 times more common in women. It is also known that the incidence of osteoarthritis increases in certain occupational groups. For example, it is more common in agricultural workers [13]. Agricultural workers have a 64% higher risk of developing osteoarthritis [14].

Mechanical loading of the knees is considered the most crucial factor in the mechanism of osteoarthritis development. Mechanical loading forms the basis of the "wear and tear" theory. In addition, in recent years, there have been publications on the genetic basis of osteoarthritis [6,15]. There is weak evidence that high estrogen levels in postmenopausal obese patients predispose them to osteoarthritis. The presence of a statistically negative relationship between smoking and the development of osteoarthritis still requires scientific explanation. Obese male patients are more likely to be associated with osteoarthritis. It is known that weight loss reduces symptoms, but it is noteworthy that this is the case only in female patients [16]. Morbid obesity does not affect surgical outcomes in patients undergoing total knee arthroplasty. However, the risk of infection is increased 6.7-fold in obese patients undergoing total knee arthroplasty [8].

After knee surgery problems, the need for an objective knee scale was recognized long ago. Although 34 new scales were proposed in the orthopedic literature from the 1970s to the 1990s, the number today is minimal. The scales that have been successful with clinicians and have continued to be used in recent years are extremely few compared with the number of recommended scales. The scales used in this study were selected from those widely used in the last 15 years and are appropriate for use in a single form [17]. Because the scales are intended for use in a single document, an attempt was made to select scales in which the patient's attention would not be distracted while completing them. It is helpful to recall the characteristics of some of the scales briefly. In particular, the WOMAC, the KSS, the OKS, and the KOOS have been used in many studies [18]. The WOMAC is a vitality scale that has been used for many years in the follow-up of elderly osteoarthritis patients. KOOS is also a scale derived from the WOMAC for follow-up in younger patients [19]. After knee surgery, 36-Item Short Form Survey (SF 36), KSS, KOOS, WOMAC, and OKS are preferred. SF 36 was not selected for developing the form in the current study because it would excessively increase the number of questions and lengthen the test duration. Even a form with seven functional and two pain scales was challenging for many patients, so no additional scales were added.

According to the study results, seven different scales for knee osteoarthritis were strongly correlated with BMI. This finding confirms the already known association between obesity and knee osteoarthritis. Although a positive correlation between osteoarthritis-related pain and obesity was predicted, the study results did not show this. Although there are publications about the positive correlation of osteoarthritis-related pain with obesity [20], our study supports the opposite. In contrast to previous scientific data, the fact that osteoarthritis-related pain was not associated with obesity in the study is a highly significant finding. However, these results need to be replicated with large and comparable groups of patients.

One of the main limitations of our article is that all patients included in the study had advanced osteoarthritis. Another limitation is that all patients were examined preoperatively, and not compared with a control group. Although at first glance, this seems to be a limitation since pain and function scales are compared, this group would probably provide more hypothesis-specific results in terms of scales. Still, on the contrary, the pain scales did not support this. Expanding the study group and including a control group in the study will increase the scientificity of such results. It is possible to obtain more meaningful results if this study is renewed by comparing preoperative and postoperative data.

## Conclusions

This study has demonstrated the association between osteoarthritis and obesity and the strong correlation with radiographic and clinical severity of knee osteoarthritis using multiple scales. However, we found that obesity was not associated with knee osteoarthritis pain. The results of this study suggest that the association between knee osteoarthritis pain and obesity still needs to be thoroughly investigated.
